# *Cobetia* sp. Bacteria, Which Are Capable of Utilizing Alginate or Waste *Laminaria* sp. for Poly(3-Hydroxybutyrate) Synthesis, Isolated From a Marine Environment

**DOI:** 10.3389/fbioe.2020.00974

**Published:** 2020-08-25

**Authors:** Hiroki Moriya, Yuto Takita, Akira Matsumoto, Yuki Yamahata, Megumi Nishimukai, Masao Miyazaki, Hitoshi Shimoi, Sung-Jin Kawai, Miwa Yamada

**Affiliations:** ^1^Department of Biological Chemistry and Food Science, Iwate University, Morioka, Japan; ^2^Department of Animal Science, Faculty of Agriculture, Iwate University, Morioka, Japan; ^3^New Field Pioneering Division, New Value Creation Center, Toyota Boshoku Corporation, Kariya, Japan; ^4^Education and Research on Sanriku Fishery Industry Department, Organization for Revitalization of the Sanriku Region and Regional Development, Iwate University, Morioka, Japan

**Keywords:** polyhydroxyalkanoate, biopolymer, seaweeds, marine biomass resources, alginic acid brown algae

## Abstract

We isolated the *Cobetia* sp. strains IU 180733JP01 (5-11-6-3) and 190790JP01 (5-25-4-2) from seaweeds and showed that both strains accumulate poly(3-hydroxybutyrate) [P(3HB)] homopolymer in a nitrogen-limiting mineral salt medium containing alginate as a sole carbon source. Genome sequence analysis of the isolated strains showed that they have putative genes which encode enzymes relevant to alginate assimilation and P(3HB) synthesis, and the putative alginate-assimilating genes formed a cluster. Investigation of the optimum culture conditions for high accumulation of P(3HB) showed that when the 5-11-6-3 strain was cultured in a nitrogen-limiting mineral salt medium (pH 5.0) containing 6% NaCl and 3% (w/v) alginate as a sole carbon source for 2 days, the P(3HB) content and P(3HB) production reached 62.1 ± 3.4 wt% and 3.11 ± 0.16 g/L, respectively. When the 5-25-4-2 strain was cultured in a nitrogen-limiting mineral salt medium (pH 4.0) containing 5% NaCl and 3% (w/v) alginate for 2 days, the P(3HB) content and P(3HB) production reached 56.9 ± 2.1 wt% and 2.67 ± 0.11 g/L, respectively. Moreover, the 5-11-6-3 strain also produced P(3HB) in a nitrogen-limiting mineral salt medium (pH 5.0) containing 6% NaCl and freeze-dried and crushed waste *Laminaria* sp., which is classified into brown algae and contains alginate abundantly. The resulting P(3HB) content and P(3HB) productivity were 13.5 ± 0.13 wt% and 3.99 ± 0.15 mg/L/h, respectively. Thus, we demonstrated the potential application of the isolated strains to a simple P(3HB) production process from seaweeds without chemical hydrolysis and enzymatic saccharification.

## Introduction

Heavy consumption of petrochemical plastic is causing serious problems in the environment all over the world. One solution to these environmental problems is the development and use of biodegradable plastics. Polyhydroxyalkanoates (PHAs) are attractive thermoplastics having biodegradability. The physical properties of PHAs closely resemble those of conventional plastics such as polypropylene and low-density polyethylene (Doi, 1990). In addition, more than 150 different structures of PHA monomers have been reported, and this diversity of monomers in PHA copolymers contributes a wide range of physical properties (Nomura and Taguchi, 2007; Tan et al., 2014; Ishii-Hyakutake et al., 2018). The PHA monomers are classified by their number of carbon atoms as short-chain-length PHAs (SCL-PHAs), medium-chain-length PHAs (MCL-PHAs), and long-chain-length PHAs (LCL-PHAs). SCL-PHAs, MCL-PHAs, and LCL-PHAs consist of 3–5 carbon atoms, 6–14 carbon atoms, and more than 14 carbon atoms, respectively (Kunasundari and Sudesh, 2011). Many bacteria produce poly(3-hydroxybutyrate) [P(3HB)], which is the most common type of PHA, and SCL and MCL-PHAs are generally produced by different types of bacteria (Taguchi and Doi, 2004).

The other main advantage of PHAs is that they can be produced by microorganisms from various substrates such as sugars, oils, and fatty acids (Sudesh et al., 2011). This property has led to the use of various kinds of biomass for PHA production as an alternative feedstock to petroleum, and thus, many researches have focused on PHA production from sugars derived from cellulosic biomass (Singh Saharan et al., 2014; Obruca, 2015). Although previous studies have identified some bacteria that produce PHA and use cellulose and lignin derivatives for growth (Tomizawa et al., 2014; Kumar et al., 2017), chemical hydrolysis and/or enzymatic saccharification of cellulosic biomass is generally required for high productivity of PHA (Obruca, 2015). Many bacteria can also utilize plant oils to accumulate PHA, and P(3HB) yields from plant oils are approximately two-fold higher than those from sugar (Akiyama et al., 2003). Thus, various plant oils (e.g., palm oil, soybean oil, olive oil, coconut oil, sunflower oil, and jatropha oil) have been evaluated as potential substrates for PHA production (Kahar et al., 2004; Ng et al., 2010). Moreover, the production of PHA from industrial and domestic wastes is an attractive approach and could help both to minimize waste disposal and to reduce the costs of PHA production. The methods of PHA production using industrial by-products such as lignocellulosic materials, molasses, fats and oils, whey, glycerol, and wastewater have been widely reported (Keenan et al., 2006; Zhu et al., 2010; Chenyu et al., 2012; Jiang et al., 2016; Scheel et al., 2019; Van Thuoc et al., 2019). However, there are few studies on PHA production from marine biomass. Thus, utilization of marine biomass can contribute to the increase in the diversity of substrates for PHA production.

Seaweeds, a component of marine biomass, have attracted attention as a foreseeable sustainable source of fuels and materials, since the marine environment represents an untapped source of energy and can supply seaweeds plentifully. For instance, several studies have reported the production of various useful compounds – biogas, ethanol, butanol, lactic acid, etc. – by fermentation with seaweed (Wise et al., 1979; Hansson, 1983; Yokoyama et al., 2007; Park et al., 2009). Seaweeds also become a focus of attention as a new substrate for PHA production. With respect to PHA production from seaweeds, studies have shown that PHA was accumulated by bacteria in a medium containing brown algae or compounds extracted from seaweed (levulinic acid) (Bera et al., 2015; Azizi et al., 2017). The red algae species *Gelidium amansii* and green macroalgae species *Ulva* have also been used for PHA production (Alkotaini et al., 2016; Sawant et al., 2018; Ghosh et al., 2019). In two of these reports (Alkotaini et al., 2016; Ghosh et al., 2019), seaweeds that were chemically hydrolyzed and/or enzymatically saccharified were used for PHA production. One-step PHA production without those pretreatments would be important for a further practical production process.

Among seaweeds, we focused on brown algae as a feedstock for PHA production. Brown algae such as Kombu (dried *Laminaria* spp.) and Wakame (*Undaria pinnatifida*) are well-reputed as foods in Japan, and huge arrays of aquaculture equipment have been erected in Japanese bays to produce these algae. However, a lot of seaweed garbage is also generated in the manufacturing, processing, and cooking of seaweed-based food products. Thus, components of brown algae such as cellulose, agar, mannitol, alginate, laminarin, carrageenan and fucoidan (Ito and Hori, 1989) have potential as good substrates for PHA production that do not compete with the production of foods. In particular, brown algae contains large amounts of mannitol (∼10 wt% in dry weight) and alginate (∼20 wt% in dry weight) (Ito and Hori, 1989). In our previous study, we isolated the *Burkholderia* sp. AIU M5M02, which produces P(3HB) from mannitol as a sole carbon source, from a marine environment (Yamada et al., 2018). At the same time, we found no PHA-production microorganism from alginate by screening. To date, the *Hydrogenophaga* sp. strain UMI-18 is the only microorganism found to produce P(3HB) from alginate as a sole carbon source (Yamaguchi et al., 2019).

In the present study, we isolated two strains, which we identified as strains of a *Cobetia* sp., that are capable of utilizing alginate as a sole carbon source for P(3HB) production and growth. The optimum culture conditions were determined to reach effective accumulation of PHA from alginate, and the metabolic pathways relevant to alginate-assimilation and P(3HB)-synthesis were predicted based on the draft genome sequence of the isolated strains. Moreover, we demonstrated that the isolated strains could produce P(3HB) from a *Laminaria* sp. without chemical hydrolysis and enzymatic saccharification treatment.

## Materials and Methods

### Isolation of the Microorganisms

The liquid culture was carried out at 30°C for 2–3 days using the Zobell Marine Broth 2216E medium containing 0.5% peptone, 0.1% yeast extract, and 0.01% FePO_4_, at pH 5.0, 7.0, or 9.0. The microorganisms grown in the medium were cultivated on an agar plate containing the Zobell Marine Broth 2216E medium at pH 5.0, 7.0, or 9.0. All isolated strains from the agar plate were cultivated again on an agar plate containing a nitrogen-limiting mineral salt (MM) medium with 1% alginate (viscosity range 300–400, FUJIFILM Wako Pure Chemical, Japan) as a sole carbon source and 0.05% Nile red at 30°C for 3 days. Strains that exhibited pinkish colonies on the agar plate containing the MM medium were selected as candidates that can produce PHA from alginate. Nile red was added from a stock solution of 25% (v/v) in dimethylsulfoxide to the agar medium at a final concentration of 0.5 μg/mL (Spiekermann et al., 1999). The MM medium (100 mL) contained 0.3% KH_2_PO_4_, 0.3% Na_2_HPO_4_, 0.05% (NH_2_)_2_CO, and 0.025% MgSO_4_⋅7H_2_O, and 1 mL of filter-sterilized trace element was added aseptically. The trace element solution consisted of (per liter) 0.22 g CoCl_2_⋅6H_2_O, 9.7 g FeCl_3_, 7.8 g CaCl_2_, 0.12 g NiCl_2_⋅6H_2_O, 0.11 g CrCl_3_⋅6H_2_O, and 0.16 g CuSO_4_⋅5H_2_O (Kahar et al., 2004).

### Identification of the Isolated Strains and Phylogenetic Analysis

The isolated strains were identified based on morphological observation, biochemical characterization (Arahal et al., 2002), and 16S rRNA analysis. Genomic DNA was extracted using a bacteria genomicPrep Mini Spin Kit (GE Healthcare United Kingdom, United Kingdom). The 16S rRNA gene was amplified by PCR using primers 16S rRNA 27F (5′-AGAGTTTGATCCTGGCTCAG-3′) and 1525R (5′-AAAGGAGGTGATCCAGCC-3′) (Weisburg et al., 1991). The PCR protocol consisted of 30 thermal cycles of 98°C for 10 s, 55°C for 30 s, and 72°C for 90 s. The similarity and identity of the sequences obtained were compared to those of other sequences in GenBank using nucleotide–nucleotide BLAST commands (Altschul et al., 1997) at the National Center for Biotechnology Information (NCBI). The phylogenetic tree base on the sequences of 16S rDNA genes was constructed using the MEGA-X software, where a neighbor-joining program was used based on the bootstrap test of 500 replicates (Felsenstein, 1985).

### PHA Biosynthesis

The isolated strains were incubated in 10 mL of MM medium containing alginate at 30°C for 2 days with shaking (120 strokes/min). The culture (3.0 mL) was inoculated into a 500-mL culture flask containing 150 mL of the MM medium containing alginate or the *Laminaria* sp. and then incubated at 30°C with shaking. Waste *Laminaria* sp. (alginate content, 6.3 wt%) was obtained from the seaweed farm in Yamada Bay (Iwate Prefecture, Japan). The alginate content of the waste *Laminaria* sp. was determined according to the previous method in the following steps (Nishide et al., 1987). The waste *Laminaria* sp. was sectioned into squares ∼10 cm on a side, and the sections were lyophilized using an FD-1000 vacuum freeze dryer (EYELA, Japan) at −80°C for 2 days. After homogenization of 10 g of the lyophilized *Laminaria* sp., 200 mL of 0.34 M Na_2_CO_3_ solution added to the slurry and the mixture was heated under stirring at 75°C for 3 h. Then, 800 mL of distilled water was added and mixed. The solution was separated from the solid matter by filtration of Celite 545 and acidified with HCl to pH 1.0. The generated precipitation was incubated at room temperature for 3 h and collected by centrifugation (3,000 *g* × 10 min, 4°C). Two hundred mL of 50% methanol was added to the precipitation, and the mixture was neutralized with NaOH under stirring. After standing overnight at room temperature, the mixture was filtered by a cotton cloth to separate the gel. The gel was washed successively with 60% methanol, 95% methanol, and acetone and was dried at 30°C for 12 h. The part of dried gel was solved to deionized water, and the concentration of alginate in the solution was measured by the Bitter–Muir method (Bitter and Muir, 1962).

With respect to the MM medium containing the waste *Laminaria* sp., the lyophilized sections were crushed into small chips. The small chips of the *Laminaria* sp. [5%(w/v)] were added to the MM medium, and the medium was autoclaved. After cultivation, the cells were harvested by centrifugation (6,400 *g* × 15 min, 4°C) and washed three times with distilled water. When the *Laminaria* sp. was used in the medium, the residue of *Laminaria* sp. was removed by a filter paper before centrifugation. The cells were then lyophilized, and the polymer was extracted with chloroform at 70°C for 48 h in glass tubes with screw caps. Cell debris was removed by passage through a PTFE filter, and then the filtrate was dried *in vacuo*. The extracted polymer was subsequently subjected to nuclear magnetic resonance (NMR), gel permeation chromatography (GPC), and differential scanning calorimetry (DSC) analyses.

### NMR, GPC, and DSC Analyses

The extracted polymers were dissolved in deuterated chloroform and analyzed by NMR. The ^1^H-NMR spectra of the polymer were obtained using a JNM-AL400 spectrometer (400 MHz; JEOL, Japan). The chemical shifts are reported in ppm, with tetramethylsilane as the internal reference. GPC and DSC analyses of extracted polymers were performed at Mitsui Chemical Analysis and Consulting Service (Japan). Polymers dissolved in hexafluoroisopropyl alcohol (HFIP) were applied to an analytical GPC (Showa Denko, Japan) equipped with Shodex HFIP-G and HFIP-606 M (Showa Denko, Japan) at 40°C. The mobile phase was HFIP containing 0.01 mM sodium trifluoroacetate. The molecular weight was estimated using a polymethyl methacrylate standard (Showa Denko, Japan). DSC data were recorded in the temperature range of −90 to 200°C on an X-DSC7000 system (Hitachi High-Tech Science, Japan) equipped with a cooling accessory under a nitrogen flow rate of 50 mL/min. The solvent-cast films (10 mg) were encapsulated in aluminum pans and heated from −90 to 200°C at 10°C/min (first heating scan). The melt samples were then rapidly quenched at −90°C and maintained at −90°C for 5 min. They were heated from −90 to 200°C at 10°C/min (second heating scan). The glass-transition temperature (*T*_g_) was taken as a midpoint of the heat capacity change. The melting temperature (*T*_m_) was determined from the positions of the endothermic peaks.

### Genome Analysis

Genomic DNA of the isolated bacterium was extracted by a bacteria genomicPrep Mini Spin Kit (GE Healthcare United Kingdom, United Kingdom). Genome sequencing, genome assembly, and gene annotation were performed at Genewiz (Japan). The genome sequence was analyzed with an Illumina HiSeq instrument (Illumina, United States). The draft genome was assembled using Velvet and gapfilled with SSPACE and GapFiller (Zerbino and Birney, 2008; Zerbino et al., 2009; Boetzer et al., 2011; Hunt et al., 2014). Prodigal (Delcher et al., 2007) gene-finding software was used to find coding genes in bacteria. The coding genes were annotated using the NCBI nr database by BLAST.

### Gas Chromatography (GC) Analysis

In order to calculate polymer content (weight percent) based on the dry cell weight and polymer productivity, GC analysis was performed. The lyophilized cells were ground into powder. By incubating ∼30 mg of lyophilized cells with 1 mL chloroform, 3.4 mL ethanol, and 0.4 mL HCl at 100°C for 4 h, P(3HB) was ethanolyzed to ethyl 3-hydroxybutyrate. Then, the esterified sample was neutralized by addition of 4 mL mixed solution (0.65 M NaOH and 0.9 M NaCl) and 2 mL solution (0.25 M Na_2_HPO_4_). The organic phase containing ethyl 3-hydroxybutyrate was mixed with 16 μg of ethyl caproate as a standard and analyzed by GC on a GC4000 Plus system (GL Science, Japan) using an HP-5 column (0.25 mm × 30 m, 0.25 μm) (Agilent, United States). The carrier gas was nitrogen at a flow rate of 1.6 mL/min. The GC conditions were as follows: an initial oven temperature of 45°C held for 1 min and increased to 80°C at a rate of 7°C/min and then to 300°C at a rate of 80°C/min, followed by a 10-min hold time.

## Results

### Isolation of PHA-Producing Bacteria From Alginate and Characterization of the Isolated Strains

Beached seaweeds of Ofunato Bay (Iwate Prefecture, Japan) were selected as sources of microorganisms. The samples were put directly into the Zobell Marine Broth 2216E medium (pH 5.0, 7.0, or 9.0), and liquid culture was carried out for 2–3 days. More than 300 colonies were isolated from the culture solution. All colonies were inoculated into the agar plate containing MM medium, Nile red, and alginate as the sole carbon source for the growth and biosynthesis of PHA, and cultured again. The Nile red-stained colonies were selected as candidates for PHA-producing microorganisms.

**FIGURE 1:**
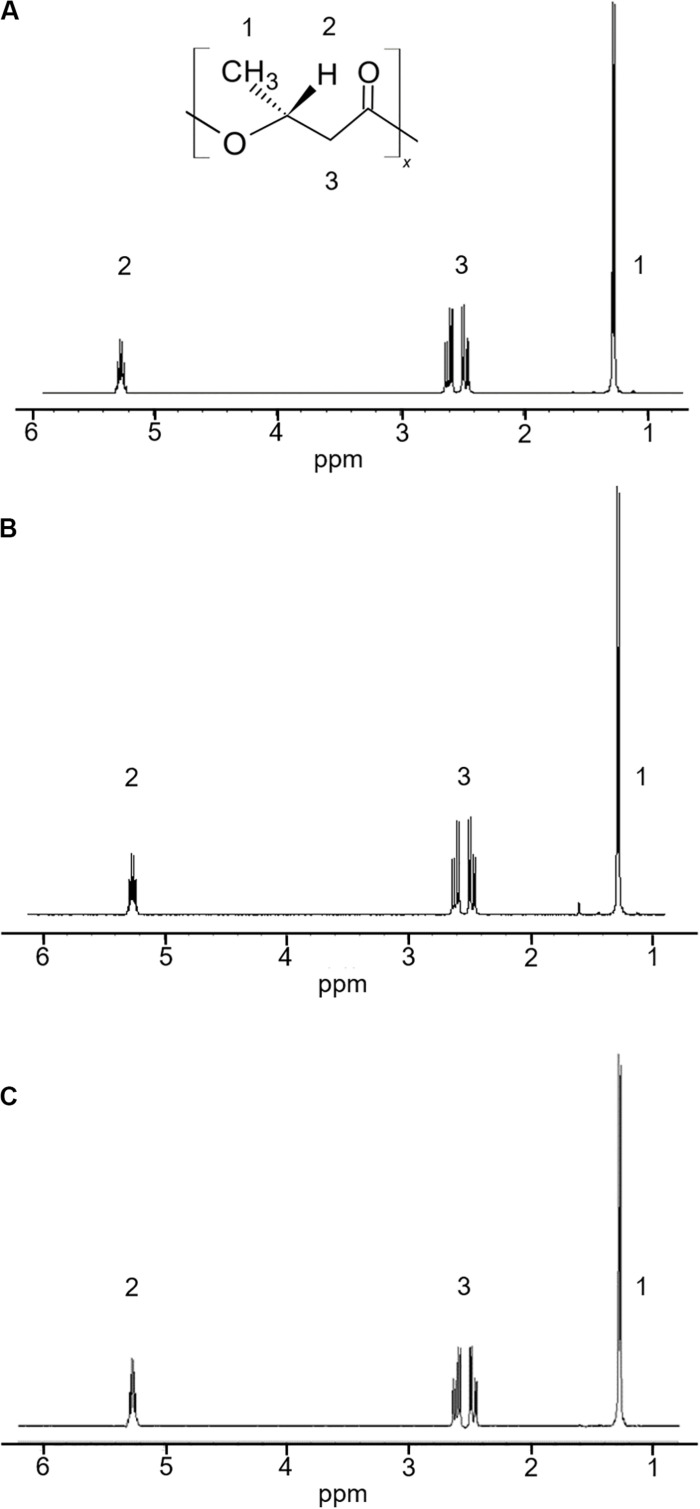
^1^H-NMR spectra of a commercial P(3HB) and P(3HB) produced from the isolated strains grown in the MM medium containing 3% (w/v) alginate at pH 5.0 and 30°C for 2 days. **(A)** P(3HB) produced from *Cobetia* sp. IU180733JP01 (5-11-6-3); **(B)** P(3HB) produced from *Cobetia* sp. IU190790JP01 (5-25-4-2); **(C)** commercial P(3HB).

The 5-11-6-3 strain and the 5-25-4-2 strain, which grew in the MM medium that contained alginate as the sole carbon source at pH 5.0, exhibited strong staining. Thus, both these strains were examined for their ability to produce PHA from alginate at 30°C for 2 days under aerobic conditions. In the ^1^H-NMR spectra, the products of the 5-11-6-3 and 5-25-4-2 strains showed the resonances for P(3HB) between 5.24 and 5.28 ppm, 2.44 and 2.64 ppm, 1.27 and 1.28 ppm (Figure 1).

### Identification of the Bacterial Strains Which Produce P(3HB) From Alginate

The 5-11-6-3 strain and the 5-25-4-2 strain were identified by phylogenetic analysis and biochemical properties (Table 1). The 16S rRNA gene sequences showed high similarity between the 5-11-6-3 strain (accession no. LC549335) and the 5-25-4-2 strain (accession no. LC549336), but they were not completely identical (99% identity, 1409/1411). Thus, we used these strains for further experiments as a different strain. The results of the 16S rRNA gene sequence of 1,411 bp from the 5-11-6-3 strain revealed a 100% identity to the partial sequence of the 16S rRNA gene of *Cobetia* sp. strain P4 (1411/1411) (accession no. MH790205); this was followed by a 100% identity to a partial sequence of the 16S rRNA gene of *Cobetia* sp. strain Aga-AMLN-15-8 (1410/1410) (accession no. MK453454). The third-closest identity was shown by the 16S rRNA gene of *Cobetia marina* strain HNS037 with 100% identity (1399/1399) (accession no. JN128271). In addition, the results of the 16S rRNA sequence of 1,435 bp from the 5-25-4-2 strain revealed a 99% identity to the partial sequence of the 16S rRNA gene of *Cobetia pacifica* strain GPM2 (1429/1433) (accession no. CP047970); this was followed by a 99% identity to a partial sequence of the 16S rRNA gene of *Cobetia* sp. strain KMM 6284 (1429/1433) (accession no. MK587632). The third-closest identity was shown by the 16S rRNA gene of *Cobetia marina* strain JCM 21022 with 99% identity (1429/1433) (accession no. NZ_CP017114).

**TABLE 1:** Characteristics of *Cobetia* sp. IU180733JP01 (5-11-6-3) and *Cobetia* sp. IU190790JP01 (5-25-4-2).

**Characteristic**	**5-11-6-3**	**5-25-4-2**
Cell shape	Straight rods	Straight rods
Motile	-	-
Gram stain	-	-
Temperature range (°C)	4-42	4-42
NaCl range (%)	0.5-20	0.5-20
Oxidase	-	-
Growth on:		
L-Arabinose	-	-
D-Glucose	+	+
Glycerol	+	+
*myo*-Inositol	+	+
Lactose	-	-
D-Mannitol	+	+
D-Mannose	+	+
D-Sorbitol	-	-
Sucrose	+	+
GC content (mol%)	62.4	62.5

*Morphological characteristics and features of the isolates were monitored on SW-5 agar plates (Arahal et al., 2002). Physiological characteristics were also determined according to Arahal et al. (2002). GC content was calculated based on the genome sequence of each strain.*

With respect to the biochemical examination of the 5-11-6-3 and 5-25-4-2 strains, the isolates were grown in D-glucose, glycerol, *myo*-inositol, D-mannitol, D-mannose, and sucrose but not L-arabinose, lactose, or D-sorbitol. In addition, the isolates were straight rod-shaped (2.0–20.0 × 0.8–1.2 μm), Gram-negative, oxidase-negative, and not motile. The isolates grew in the temperature range from 4 to 42°C and in the NaCl range from 0.5 to 20%. These properties were almost the same as those of *Cobetia marina* and *Cobetia pacifica*, but *C. marina* and *C. pacifica* can utilize L-arabinose but not D-mannose for growth (Romanenko et al., 2013). Thus, the 5-11-6-3 and 5-25-4-2 strains were identified as strains of *Cobetia* sp. according to all of the identification results. The isolates were deposited in the National Institute of Technology and Evaluation (NITE). The code names were *Cobetia* sp. IU180733JP01 (5-11-6-3) (NITE P-02758) and *Cobetia* sp. IU190790JP01 (5-25-4-2) (NITE P-03085), respectively.

**FIGURE 2:**
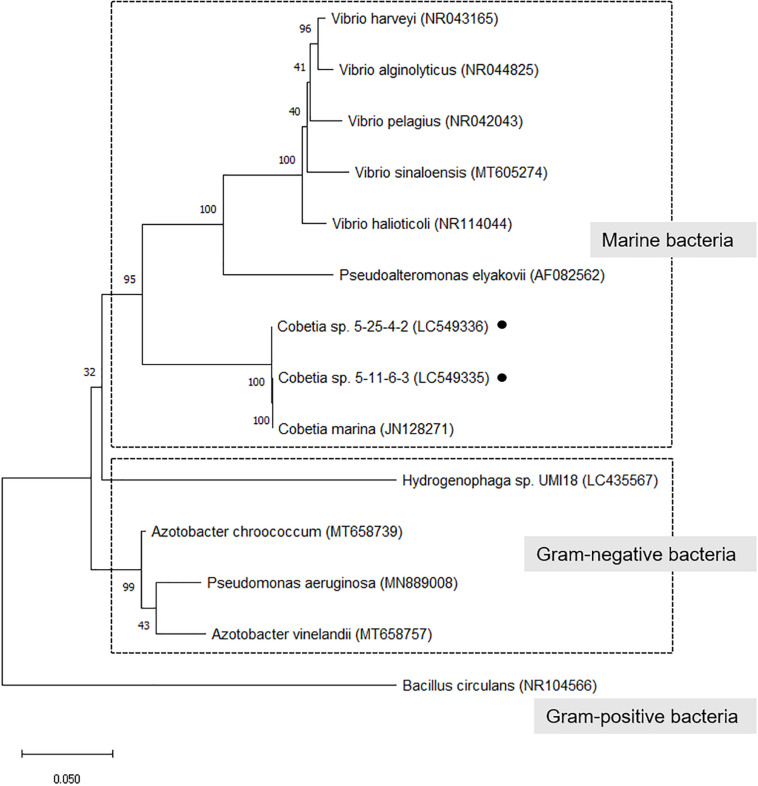
Phylogenetic tree of isolated strains with alginate-degrading bacteria (alginate lyase-producing bacteria). The trees were constructed by the neighbor-joining method with MEGA-X software. The GenBank accession no. was described after each microorganism name. The numbers at the branch nodes represent the levels of bootstrap support based on the analyses of 500 replicates. Bar 0.05 substitutions per site.

In a phylogenetic tree of isolated strains with alginate-degrading bacteria (alginate lyase-producing bacteria) (Wong et al., 2000; Yamaguchi et al., 2019), the isolated strains closely related to *C. marina* and belonged to a cluster of marine bacteria (Figure 2). However, there are no reports that these bacteria except *Hydrogenophaga* sp. UMI-18 exhibited PHA production from alginate.

### Genome Analysis of the Isolated Strains and Prediction of the Alginate-Assimilating and P(3HB) Biosynthesis Pathways

The assembled genome of the 5-11-6-3 strain and 5-25-4-2 strain consisted of 40 scaffolds with from 281 to 925,956 bp (accession nos. BLWJ01000001–BLWJ01000040) and 41 scaffolds with from 261 to 1,114,877 bp (accession nos. BLWK01000001–BLWK01000041), respectively. According to the results of assembly between the genome sequences of the 5-11-6-3 strain and the 5-25-4-2 strain, the two genomes were closely related, but the 5-11-6-3 strain and 5-25-4-2 strain were different strains.

**FIGURE 3:**
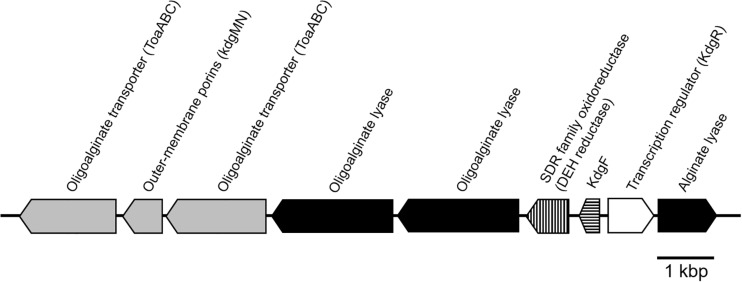
Schematic representation of the putative gene cluster encoding enzymes relevant to alginate assimilation in *Cobetia* sp. IU180733JP01 (5-11-6-3) and *Cobetia* sp. IU190790JP01 (5-25-4-2). The gene cluster was 13.5 kbp. Oligoalginate transporter and outer membrane porins genes: gray; alginate lyase genes, black; DEH reductase gene, vertical stripe; Kdg F, horizontal stripe; transcription regulator, white.

**FIGURE 4:**
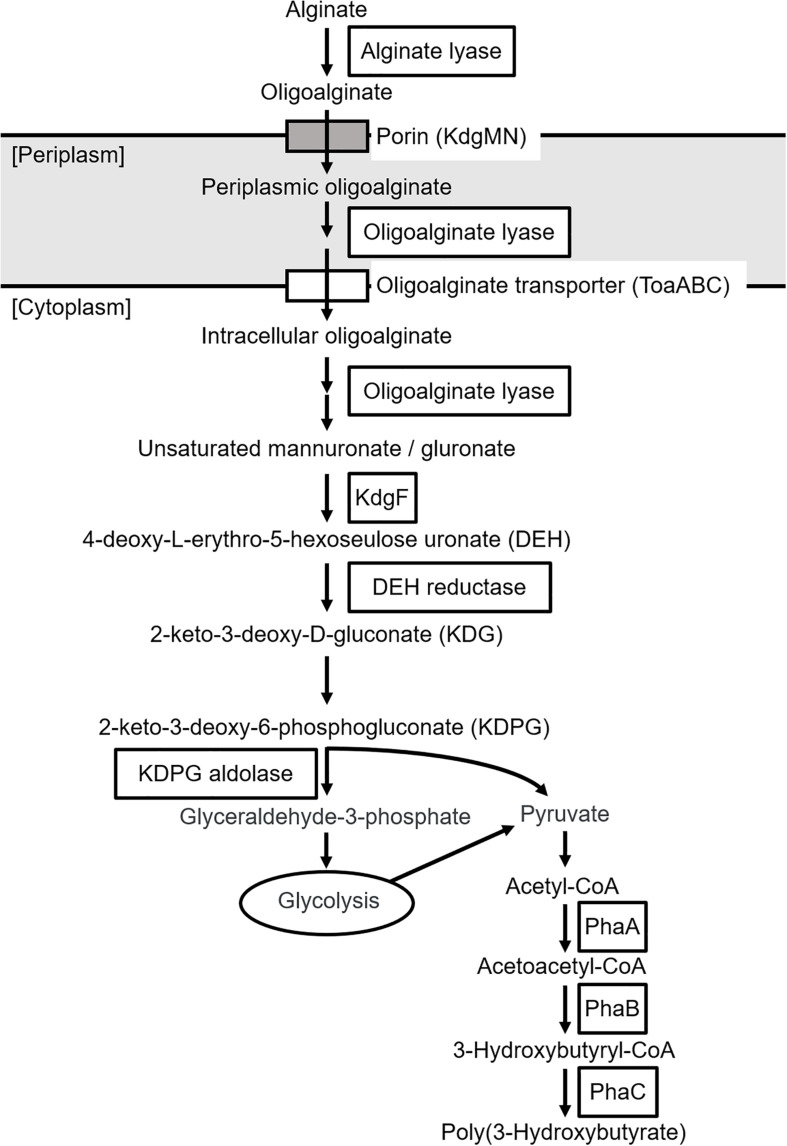
Predicted biosynthetic pathway of P(3HB) from alginate in *Cobetia* sp. IU180733JP01 (5-11-6-3) and *Cobetia* sp. IU190790JP01 (5-25-4-2). PhaA, β-ketothiolase; PhaB, acetoacetyl-coenzyme A reductase; PhaC, PHA synthase.

The putative genes encoding the proteins or enzymes related to alginate assimilation, such as alginate lyase (Yagi et al., 2016), oligoalginate transporters (ToaABC) (Kawai and Murata, 2016), outer-membrane porins (KdgMN) of oligo alginate (Kawai and Murata, 2016), oligoalginate lyase (Yagi et al., 2016), SDR family oxidoreductase (DEH reductase) (Inoue et al., 2015), and Kdgf which converts unsaturated mannuronate and gluronate into 4-deoxy-L-erythro-5-hexoseulose uronate (DEH) (Hobbs et al., 2016), were located in one cluster found in scaffold 8 (301,144 bp) of the 5-11-6-3 strain and in scaffold 6 (306,581 bp) of the 5-25-4-2 strain (Tables 2, 3 and Figure 3). These enzymes would constitute the metabolic pathway to convert alginate to DEH in the 5-11-6-3 and 5-25-4-2 strains (Figure 4). Furthermore, the putative gene encoding 2-keto-3-deoxy-6-phosphogluconate (KDPG) aldolase, which catalyzes the cleavage of KDPG to glyceraldehyde-3-phosphate and pyruvate (Nishiyama et al., 2017), was found in scaffold 1 (925,956 bp) of the 5-11-6-3 strain and in scaffold 1 (1,114,691 bp) of the 5-25-4-2 strain. However, the putative gene encoding 2-keto-3-deoxy-D-gluconate (KDG) kinase, which converts KDG to KDPG, was not found in the draft genome sequences of either strain.

**TABLE 2:** Putative genes encoding the proteins or enzymes related to alginate assimilation and P(3HB) synthesis in the draft genome of *Cobetia* sp. IU180733JP01 (5-11-6-3) (accession nos. BLWJ01000001–BLWJ01000040).

**Scaffold**	**Gene length (bp)**	**Description**	**Accession no.**	**Locus tag number**
8	1,059	Polysaccharide lyase family 7 protein (alginate lyase)	BLWJ01000008	Cobetia51163_080091
8	801	DNA-binding transcriptional regulator KdgR	BLWJ01000008	Cobetia51163_080092
8	345	KdgF	BLWJ01000008	Cobetia51163_080093
8	774	SDR family oxidoreductase	BLWJ01000008	Cobetia51163_080094
8	2,160	Oligoalginate lyase	BLWJ01000008	Cobetia51163_080095
8	2,142	Oligoalginate lyase	BLWJ01000008	Cobetia51163_080096
8	1,788	Transporter ToaABC-1	BLWJ01000008	Cobetia51163_080097
8	693	Outer-membrane porins (kdgMN)	BLWJ01000008	Cobetia51163_080098
8	1,716	Transporter ToaABC-2	BLWJ01000008	Cobetia51163_080099
1	663	2-Dehydro-3-deoxy-phosphogluconate aldolase	BLWJ01000001	Cobetia51163_010600
1	1,179	Acetyl-CoA C-acyltransferase (PhaA)	BLWJ01000001	Cobetia51163_010751
2	1,221	Acetyl-CoA C-acyltransferase (PhaA)	BLWJ01000002	Cobetia51163_020365
5	1,182	Acetyl-CoA acetyltransferase (PhaA)	BLWJ01000005	Cobetia51163_050288
13	747	Acetoacetyl-CoA reductase (PhaB)	BLWJ01000013	Cobetia51163_130024
1	2,829	Class I poly(*R*)-hydroxyalkanoic acid synthase (PhaC)	BLWJ01000001	Cobetia51163_010347

*Nucleotide sequences of the genes are available in DDBJ/EMBL/GenBank with the accession numbers and their locus tag numbers.*

**TABLE 3:** Putative genes encoding the proteins or enzymes related to alginate assimilating and P(3HB) synthesis in the draft genome of *Cobetia* sp. IU 190790JP01 (5-25-4-2) (accession nos. BLWK01000001–BLWK01000041).

**Scaffold**	**Gene length (bp)**	**Description**	**Accession no.**	**Locus tag number**
6	1,716	Transporter ToaABC-2	BLWK01000006	Cobetia52542_060124
6	693	Outer-membrane porins (KdgMN)	BLWK01000006	Cobetia52542_060125
6	1,788	Transporter ToaABC-1	BLWK01000006	Cobetia52542_060126
6	2,142	Oligoalginate lyase	BLWK01000006	Cobetia52542_060127
6	2,160	Oligoalginate lyase	BLWK01000006	Cobetia52542_060128
6	774	SDR family oxidoreductase	BLWK01000006	Cobetia52542_060129
6	345	Alginate and pectin degradation protein (Kdgf)	BLWK01000006	Cobetia52542_060130
6	801	DNA-binding transcriptional regulator (KdgR)	BLWK01000006	Cobetia52542_060131
6	1,059	Polysaccharide lyase family 7 protein	BLWK01000006	Cobetia52542_060132
1	663	2-Dehydro-3-deoxy-phosphogluconate aldolase	BLWK01000001	Cobetia52542_010170
1	1,179	Acetyl-CoA C-acyltransferase (PhaA)	BLWK01000001	Cobetia52542_010019
3	1,221	Acetyl-CoA C-acyltransferase (PhaA)	BLWK01000003	Cobetia52542_030022
4	1,182	Acetyl-CoA C-acyltransferase (PhaA)	BLWK01000004	Cobetia52542_040024
12	747	Acetoacetyl-CoA reductase (PhaB)	BLWK01000012	Cobetia52542_120023
1	3,018	Class I poly(*R*)-hydroxyalkanoic acid synthase (PhaC)	BLWK01000001	Cobetia52542_010422

*Nucleotide sequences of the genes are available in DDBJ/EMBL/GenBank with the accession numbers and their locus tag numbers.*

**FIGURE 5:**
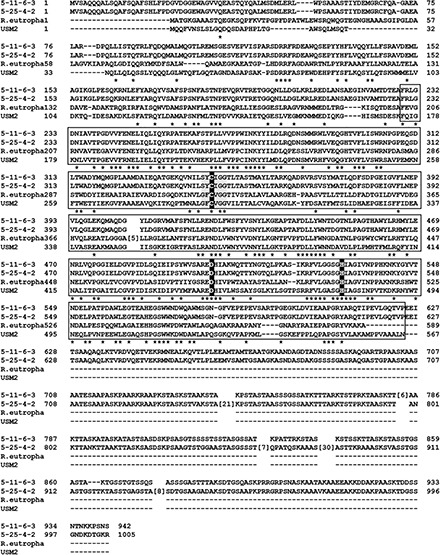
Multiple alignments of putative Class I PHA synthase (PhaC) from *Cobetia* sp. IU180733JP01 (5-11-6-3) and *Cobetia* sp. IU190790JP01 (5-25-4-2) with PhaC from *Ralstonia eutropha* (accession no. WP_078196023) and *Chromobacterium* sp. USM2 (accession no. ADL70203). Asterisks, identical residues; white box, α/β hydrolase domain of PhaC from *R. eutropha* and *Chromobacterium* sp. USM2; black box, catalytic triad residues; gray box, lipase-like box residues. The multiple alignments were performed with Constraint-based Multiple Alignment Tool (COBALT) (Papadopoulos and Agarwala, 2007) at NCBI.

The putative genes encoding the enzymes related to P(3HB) synthesis were also found in the genome of the 5-11-6-3 and 5-25-4-2 strains, but they were not located in the same cluster. Three putative genes encoding β-ketothiolase (PhaA), which catalyzes the condensation of two molecules of acetyl-CoA to form acetoacetyl-CoA (Sudesh et al., 2000), were found in scaffold 1 (925,956 bp), 2 (480,608 bp), and 5 (355,745 bp) of the 5-11-6-3 strain (Tables 2, 3 and Figure 4). In the genome sequence of the 5-25-4-2 strain, there are also three putative genes encoding PhaA in scaffolds 1 (1,114,691 bp), 3 (485,930 bp), and 4 (343,977 bp). The putative genes encoding other enzymes required for P(3HB) synthesis, such as NADPH-dependent acetoacetyl CoA reductase (PhaB) and Class I PHA synthase (PhaC) (Sudesh et al., 2000), were present in scaffolds 13 (53,722 bp) and 1 (925,956 bp) of the 5-11-6-3 strain, respectively. In the 5-25-4-2 strain, the putative genes encoding PhaB and PhaC were found in scaffolds 12 (52,686 bp) and 1 (1,114,691 bp), respectively. We compared the putative amino acid sequences of PhaC from the 5-11-6-3 and 5-25-4-2 strains with sequences of PhaC from *Ralstonia eutropha* and *Chromobacterium* sp. USM2, whose crystal structures were successfully determined (Wittenborn et al., 2016; Chek et al., 2017, 2019, 2020; Kim et al., 2017; Figure 5). The amino acid sequences were relatively conserved around the α/β hydrolase domain. The conserved catalytic triad residues and lipase-like box residues were also found in the putative PhaC from the 5-11-6-3 and 5-25-4-2 strains.

All putative genes related to alginate assimilation and P(3HB) synthesis listed in Tables 2, 3 with the exception of *phaC* showed over 99% identity between the 5-11-6-3 strain and 5-25-4-2 strain, while only the putative gene encoding PhaC did not show high identity between the 5-11-6-3 strain and 5-25-4-2 strain (Supplementary Figure S1). The presence of an alginate-assimilating gene cluster and P(3HB)-synthesis genes in the genome of the 5-11-6-3 and 5-25-4-2 strains supported that the 5-11-6-3 and 5-25-4-2 strains have the ability to synthesize P(3HB) from alginate as a sole carbon source.

### Effects of Alginate Concentration, pH, and NaCl Concentration on P(3HB) Biosynthesis

**FIGURE 6:**
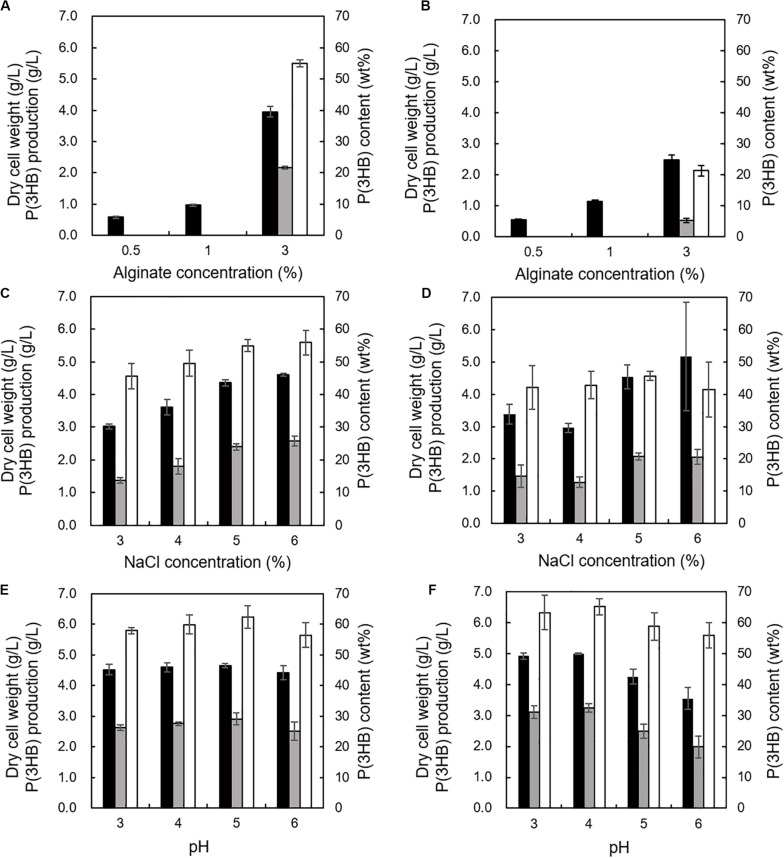
Effects of alginate concentration, NaCl concentration, and pH on growth and P(3HB) production in *Cobetia* sp. IU180733JP01 (5-11-6-3) and *Cobetia* sp. IU190790JP01 (5-25-4-2). **(A)** Effect of alginate concentration on growth, P(3HB) production, and P(3HB) content of *Cobetia* sp. IU180733JP01 (5-11-6-3) in the MM medium containing 2% NaCl at 30°C, pH 5.0 for 3 days. **(B)** Effect of alginate concentration on growth, P(3HB) production, and P(3HB) content of *Cobetia* sp. IU190790JP01 (5-25-4-2) in the MM medium containing 2% NaCl at 30°C, pH 5.0, for 2 days. **(C)** Effects of NaCl concentration on growth, P(3HB) production, and P(3HB) content of *Cobetia* sp. IU180733JP01 (5-11-6-3) in the MM medium containing 3% (w/v) alginate at 30°C, pH 5.0 for 3 days. **(D)** Effect of NaCl concentration on growth, P(3HB) production, and P(3HB) content of *Cobetia* sp. IU190790JP01 (5-25-4-2) in the MM medium containing 3% (w/v) alginate at 30°C, pH 5.0, for 2 days. **(E)** Effects of pH on growth, P(3HB) production, and P(3HB) content of *Cobetia* sp. IU180733JP01 (5-11-6-3) in the MM medium containing 3% (w/v) alginate and 6% NaCl at 30°C for 3 days. **(F)** Effect of pH on growth, P(3HB) production, and P(3HB) content of *Cobetia* sp. IU190790JP01 (5-25-4-2) in the MM medium containing 3% (w/v) alginate and 5% NaCl at 30°C for 2 days. Closed bars, dry cell weight; gray bars, P(3HB) production; open bars, P(3HB) content. Dry cell weight was measured after freeze-drying. The P(3HB) content of freeze-dried cells was determined by the weight of the produced P(3HB). The data represent means ± S.D (*n* = 3).

The effects of alginate concentrations between 0.5 and 3% in the MM medium with 2% NaCl were investigated at 30°C and pH 5.0 using the 5-11-6-3 strain or the 5-25-4-2 strain. We determined the cultivation temperature (30°C) according to the previous reports which evaluated PHA productivity using other PHA-producing microorganisms such as *R. eutropha*, *Pseudomonas putida*, recombinant *Escherichia coli*, and *Hydrogenophaga* sp. UMI-18 (Fidler and Dennis, 1992; Kichise et al., 1999; Wang and Nomura, 2010; Yamaguchi et al., 2019). The 3% (w/v) alginate concentration was suitable for well growth and high P(3HB) content and P(3HB) production of both strains (Figures 6A,B). However, when the alginate concentration was increased to more than 3% in the medium, it became difficult to prepare the medium due to the high viscosity. Next, the effect of NaCl concentration on P(3HB) production was evaluated in the MM medium containing 3% (w/v) alginate at 30°C and pH 5.0. In the NaCl concentration range from 3 to 6%, the cell growth, P(3HB) content, and P(3HB) production of the 5-11-6-3 strain increased along with the NaCl concentration (Figure 6C). However, alginate was deposited when the NaCl concentration was more than 6% in the medium, and we were not able to examine the culture conditions using the medium containing more than 6% NaCl. The 5-25-4-2 strain exhibited good growth in the medium containing 6% NaCl, but maximum P(3HB) content and P(3HB) production were confirmed in the medium containing 5% NaCl (Figure 6D). Subsequently, we investigated the effect of pH on PHA production. The 5-11-6-3 strain and the 5-25-4-2 strain were not able to grow at pH 2. In addition, 3% (w/v) alginate was deposited in the medium at higher than pH 7. Thus, we examined the optimum pH in the range from 3 to 6. When the 5-11-6-3 strain was cultured in the MM medium containing 3% (w/v) alginate and 6% NaCl at 30°C, there was no significant effect on the cell growth, P(3HB) content, or P(3HB) production in the pH range from 4 to 6 (Figure 6E). The P(3HB) production at pH 5.0 was slightly higher than that at other pH ranges. When the 5-25-4-2 strain was cultured in the MM medium containing 3% (w/v) alginate and 5% NaCl at 30°C, the cell growth, P(3HB) content, and P(3HB) production were highest at pH 4.0 (Figure 6F). We therefore further investigated the time course of growth and P(3HB) production of isolated strains using the MM medium containing 3% (w/v) alginate and 6% NaCl at pH 5.0 (for the 5-11-6-3 strain), or 3% (w/v) alginate and 5% NaCl at pH 4.0 (for the 5-25-4-2 strain).

### Growth Profile and P(3HB) Accumulation Under Optimized Culture Conditions

**FIGURE 7:**
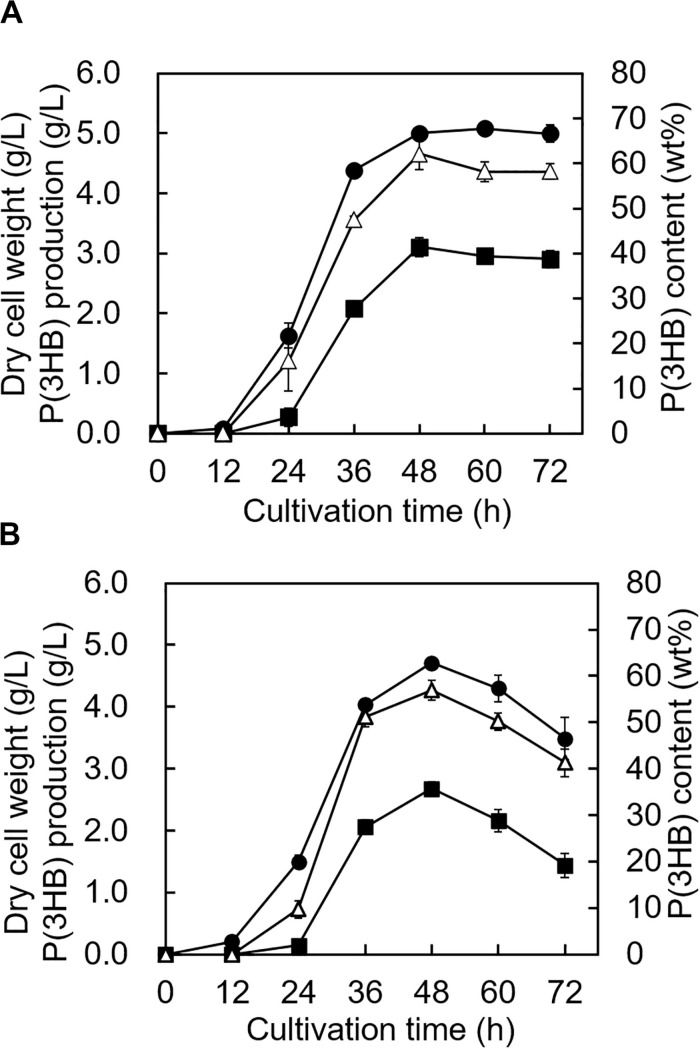
Time profiles of growth and P(3HB) synthesis by *Cobetia* sp. IU180733JP01 (5-11-6-3) and *Cobetia* sp. IU190790JP01 (5-25-4-2). **(A)** *Cobetia* sp. IU180733JP01 (5-11-6-3) was cultured in the MM medium containing 3% (w/v) alginate and 6% NaCl at pH 5.0 and 30°C. **(B)** *Cobetia* sp. IU190790JP01 (5-25-4-2) was cultured in the MM medium containing 3% (w/v) alginate and 5% NaCl at pH 4.0 and 30°C. Filled circles, dried cell weight; opened triangles, P(3HB) content; closed squares, P(3HB) production. The data represent means ± S.D (*n* = 3). Dry cell weight was measured after freeze-drying. The P(3HB) content of freeze-dried cells was determined by the weight of the produced P(3HB).

The growth profile and P(3HB) accumulation of the 5-11-6-3 strain and 5-25-4-2 strain were investigated in the MM medium containing the optimum alginate concentration, NaCl concentration, and pH at 30°C. The growth profile of the 5-11-6-3 strain showed that the cells grew rapidly until 48 h, and the cell biomass increased gradually from 48 to 72 h (Figure 7A). During the period of exponential growth, the P(3HB) content and production also increased significantly, but the P(3HB) accumulation was almost saturated after 48 h of cultivation. After 48 h of cultivation, the maximum P(3HB) content and P(3HB) production were 62.1 ± 3.4 wt% and 3.11 ± 0.16 g/L, respectively. The cell biomass, P(3HB) content, and P(3HB) production of the 5-25-4-2 strain also grew rapidly until 48 h, but the cell biomass, P(3HB), and P(3HB) production decreased after 48 h (Figure 7B). The maximum P(3HB) content and P(3HB) production were 56.9 ± 2.1 wt% and 2.67 ± 0.11 g/L, respectively, at 48 h of cultivation.

The molecular weights of P(3HB) obtained from the 5-11-6-3 strain under optimum culture conditions were determined to be *M*n = 20.5 × 10^4^ and *M*w = 108 × 10^4^, respectively, using analytical GPC (Table 4). The molecular weights of P(3HB) obtained from the 5-25-4-2 strain under optimum culture conditions were determined to be *M*n = 24.7 × 10^4^ and *M*w = 117 × 10^4^, respectively (Supplementary Figure S2). According to the DSC analysis, glass-transition temperature (*T*_g_) and melting temperature (*T*_m_) values for the P(3HB) obtained from the 5-11-6-3 strain were determined as 5 and 177.4°C, respectively (Table 4). The *T*_g_ and *T*_m_ values for P(3HB) obtained from the 5-25-4-2 were 5.1 and 176.0°C, respectively.

**TABLE 4:** Thermal properties and molecular weights of P(3HB) produced by *Cobetia* sp. IU180733JP01 (5-11-6-3) and *Cobetia* sp. IU190790JP01 (5-25-4-2) from alginate.

**Polymer**	***T*_g_ (°C)**	***T*_m_ (°C)**	***M*_w_ (× 10^4^)**	***M*_n_ (× 10^4^)**	***M*_w_/*M*_n_**	**References**
5-11-6-3	5.0	177.4	108	20.5	5.2	This study
5-25-4-2	5.1	176.0	117	24.7	4.7	This study
Commercial P(3HB)	3.5	175.4	97	33	2.9	(Tanadchangsaeng and Yu, 2012)

*Cobetia sp. IU180733JP01 (5-11-6-3) was cultured in the MM medium containing 3% (w/v) alginate, 6% NaCl at pH 5.0, and 30°C for 48 h. Cobetia sp. IU190790JP01 (5-25-4-2) was cultured in the MM medium containing 3% (w/v) alginate, 5% NaCl, at pH 4.0, and 30°C for 48 h.*

Although the maximum P(3HB) production and polymer properties showed no significant differences between the 5-11-6-3 strain and the 5-25-4-2 strain, the 5-11-6-3 strain continued to exhibit high P(3HB) accumulation after 48 h of cultivation. Thus, we selected the 5-11-6-3 strain for further experiments.

### Biosynthesis of P(3HB) From Waste *Laminaria* sp. Using *Cobetia* sp. IU180733JP01 (5-11-6-3)

**FIGURE 8:**
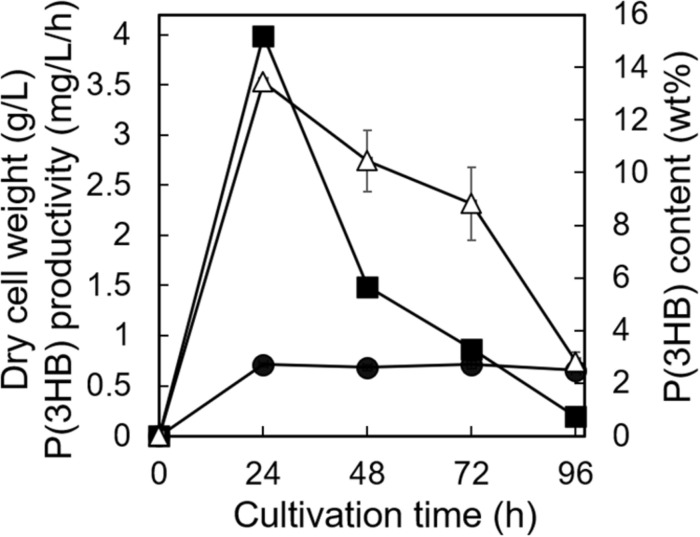
Time profiles of growth and P(3HB) synthesis by *Cobetia* sp. IU180733JP01 (5-11-6-3) from waste *Laminaria* species. *Cobetia* sp. IU180733JP01 (5-11-6-3) was cultured in the MM medium containing 6% NaCl and 5% (w/v) *Laminaria* sp. at pH 5.0 and 30°C. After *Laminaria* sp. was lyophilized and crushed into small chips, the small chips *Laminaria* sp. were added to the MM medium. Filled circles, dried cell weight; opened triangles, P(3HB) content; closed squares, P(3HB) productivity. The data represent means ± S.D (*n* = 3). Dry cell weight was measured after freeze-drying. The P(3HB) content of freeze-dried cells was determined by the weight of the produced P(3HB).

The utilization of low-purity substrates for P(3HB) production is advantageous for industrial application. We tried to use waste *Laminaria* sp. obtained from a seaweed farm in Yamada Bay (Iwate Prefecture, Japan) for P(3HB) production. The growth profile and P(3HB) accumulation of the 5-11-6-3 strain were evaluated in the MM medium containing 6% NaCl and 5%(w/v) freeze-dried and crushed *Laminaria* sp. at pH 5.0 for 4 days (Figure 8). After 24 h of cultivation, the cell growth was saturated and P(3HB) accumulation was confirmed [the P(3HB) content and P(3HB) productivity were 13.5 ± 0.13 wt% and 3.99 ± 0.15 mg/L/h, respectively]. The P(3HB) content and P(3HB) productivity decreased gradually after 24 h. In addition, after 4 days of cultivation, weight of seaweeds was decreased to approximately 45 wt% of the adding amount before cultivation.

## Discussion

In order to utilize untapped bioresources for PHA production, we searched for microorganisms that produce PHA from alginate, which is an abundant component of seaweed, as a sole carbon source in the culture medium. By screening environmental microorganisms using Nile red staining, we succeeded in isolating two strains, *Cobetia* sp. IU180733JP01 (5-11-6-3) and *Cobetia* sp. IU190790JP01 (5-25-4-2). In our previous study, we also searched a marine environment for a microorganism that can use either alginate or mannitol for PHA production. However, we found only *Burkholderia* sp. AIU M5M02, which is capable of producing P(3HB) in the culture medium containing mannitol as a sole carbon source (Yamada et al., 2018). There were two differences in the screening process between the previous study and our present one. The first difference was the source of the isolated microorganisms. Although in our previous screening we isolated microorganisms from shallow sea mud samples taken from Ofunato Bay, in this study we used beached seaweeds obtained from Ofunato Bay as a source of microorganisms. Another difference concerned the components of the culture medium used for microorganism isolation. In our previous screening, the samples were inoculated directly into the MM medium containing alginate or mannitol as the sole carbon source in order to enrich target microorganisms. In this study, we used Zobell Marine Broth 2216E medium, which is suitable for culturing marine bacteria, for isolation. Thus, our finding of new microorganisms having the ability to biosynthesize P(3HB) from alginate would be due to these differences in the screening process.

To our knowledge, there has been no report about microorganisms that can produce PHA from alginate as a sole carbon source, except for *Hydrogenophaga* sp. UMI-18 (Yamaguchi et al., 2019). We therefore predicted the metabolic pathways relevant to alginate assimilation and P(3HB) biosynthesis in the isolated strains by genome analysis. In the predicted pathways, alginate is converted to pyruvate, a precursor of acetyl-CoA via glycolysis, and P(3HB) is synthesized from acetyl-CoA by three enzyme reactions (Figure 4). This predicted P(3HB) synthesis pathway from alginate in the isolated strains was similar to the predicted pathway in *Hydrogenophaga* sp. strain UMI-18. However, unlike in *Hydrogenophaga* sp. strain UMI-18, the putative gene encoding KDPG aldolase was not located in an alginate-assimilating gene cluster, and the putative genes encoding enzymes which catalyze conversion reactions from acetyl-CoA to P(3HB) did not form one gene cluster.

We investigated the optimum culture conditions, such as the alginate concentration, NaCl concentration, pH of the culture medium, and culture time, for high P(3HB) accumulation in the isolated strains. The ability to accumulate P(3HB) was almost identical between the 5-11-6-3 strain and the 5-25-4-2 strain, while the 5-25-4-2 strain showed a decrease in P(3HB) content and P(3HB) production after 48 h of cultivation (Figure 7). This rapid degradation of P(3HB) in the 5-25-4 strain may be due to differences derived from P(3HB) depolymerase (PhaZ) between the 5-11-6-3 strain and the 5-25-4-2 strain. However, the putative genes encoding PhaZ were not found in our homology search. Thus, it would be expected that other enzymes played a role of PhaZ in the 5-11-6-3 strain and the 5-25-4-2. Differences of those enzymes may lead the rapid degradation of P(3HB) in the 5-25-4 strain. When *Hydrogenophaga* sp. UMI-18 was cultured in mineral salt medium containing alginate, the maximum P(3HB) content was 58 ± 4 wt% (Yamaguchi et al., 2019). This value was lower than the maximum P(3HB) content of the 5-11-6-3 strain (62.1 ± 3.4 wt%) and was similar to the maximum P(3HB) content of the 5-25-4-2 strain (56.9 ± 2.1 wt%). Precise comparison of the P(3HB) accumulation abilities between the isolated strains and *Hydrogenophaga* sp. UMI-18 was difficult because of the experimental differences, such as the culture scale and culture manipulation, but the abilities of our isolated strains to accumulate P(3HB) were suggested to be similar to or higher than that of *Hydrogenophaga* sp. UMI-18. Moreover, the molecular weights and thermal properties of P(3HB) produced by the isolated strains were comparable to those of commercial P(3HB) (Table 4), which is advantageous for practical PHA production. The broad polydispersity for P(3HB)s produced by the 5-11-6-3 strain and 5-25-4-2 strain would be due to a nature of their PhaCs, the amount of PhaC, and the simultaneous degradation of P(3HB) during biosynthesis in the cell. It is known that these factors affect the molecular weight and the polydispersity of PHA in the cell (Tsuge, 2016).

When waste *Laminaria* sp. was added to the medium, the P(3HB) content and production of the 5-11-6-3 strain were lower than those when 3% (w/v) alginate was added (Figures 7, 8). Moreover, the P(3HB) content rapidly decreased after the cells reached to the stationary phase in the cultivation using waste *Laminaria* sp. We presumed that there would be two reasons: one is shortage of carbon source because the concentration of alginate in medium containing waste *Laminaria* sp. was approximately 0.3% (w/v). Another possibility is that the C/N ratio in the medium containing waste *Laminaria* sp. would not be appropriate to PHA production. It is well known that the C/N ratio of the medium also affects PHA productivity (Faccin et al., 2009; Yang et al., 2010). Considering the rapid decrease in P(3HB) content in the stationary phase, the former would be a main cause in this experimental culture conditions. In order to understand in detail, we should clarify which kinds of carbon sources of waste *Laminaria* sp. would be easily utilized for growth and P(3HB) production by the 5-11-6-3 strain in our future work.

Some researches have focused on waste or inedible seaweeds such as red algae *Gelidium amansii* (Alkotaini et al., 2016; Sawant et al., 2018), green macroalgae *Ulva* sp. (Ghosh et al., 2019), and brown algae *Sargassum* sp. (Azizi et al., 2017) as a raw material of PHA production. In most of these studies, the seaweed was subjected to chemical hydrolysis and/or enzymatic saccharification before cultivation. If a process could be established that includes hydrolysis and fermentation in a single step, this would be a promising approach for producing PHA from marine biomass. Recently, Sawant et al. succeeded in producing *Saccharophagus degradans* 2–40 P(3HB) from red algae without pretreatment (Sawant et al., 2018). In the cultivation of *S. degradans* 2–40 using the medium including untreated red algae, the P(3HB) content was from 12 to 16 wt%, which was a similar value to the P(3HB) content of our isolated strain using the MM medium including *Laminaria* sp. without chemical hydrolysis and enzymatic saccharification. Furthermore, *S. degradans* 2–40 required at least 65 h of cultivation to reach 12 wt% of P(3HB) content. Our isolated strain accumulated an amount of P(3HB) almost equal to that accumulated by *S. degradans* 2–40 (13.5 ± 0.13 wt%) by culture of only 24 h. Thus, our isolated strain is an appropriate candidate for application to P(3HB) production from not only pure alginate extracted from seaweeds but also brown algae. In order to enhance P(3HB) productivity using the isolated strain and brown algae, it will be important to fine tune the culture conditions in our future experiments.

## Data Availability Statement

The datasets presented in this study can be found in online repositories. The names of the repositories and accession numbers can be found below: https://www.ncbi.nlm.nih.gov/genbank/, LC549335; https://www.ncbi.nlm.nih.gov/genbank/, LC549336; https://www.ncbi.nlm.nih.gov/genbank/, BLWJ01000008; https://www.ncbi.nlm.nih.gov/genbank/, BLWJ01000001; https://www.ncbi.nlm.nih.gov/genbank/, BLWJ01000002; https://www.ncbi.nlm.nih.gov/genbank/, BLWJ01000005; https://www.ncbi.nlm.nih.gov/genbank/, BLWJ01000013; https://www. ncbi.nlm.nih.gov/genbank/, BLWK01000006; https://www.ncbi. nlm.nih.gov/genbank/, BLWK01000001; https://www.ncbi.nlm. nih.gov/genbank/, BLWK01000003; https://www.ncbi.nlm.nih.gov/genbank/, BLWK01000004; https://www.ncbi.nlm.nih.gov/genbank/, BLWK01000012.

## Author Contributions

MY designed the study and revised the manuscript. HM, YT, and MY wrote the manuscript with the help of MM, MN, HS, and S-JK. HM, YT, AM, and YY performed the experiments with the help of MN and MM. MY, HS, and S-JK analyzed the results. All authors viewed and approved the manuscript and contributed significantly to the work.

## Conflict of Interest

S-JK was employed by the company Toyota Boshoku Corporation. The remaining authors declare that the research was conducted in the absence of any commercial or financial relationships that could be construed as a potential conflict of interest.
