# Supplementary material for: Cobetia sp. Bacteria, Which Are Capable of Utilizing Alginate or Waste Laminaria sp. for Poly(3-Hydroxybutyrate) Synthesis, Isolated From a Marine Environment
Source: Front Bioeng Biotechnol. 2020 Aug 25;8:974. doi: 10.3389/fbioe.2020.00974 (PMC7479843; doi:10.3389/fbioe.2020.00974)
Supplement: Supplementary file 1 [file Data_Sheet_1.DOCX]

**Supplementary Material**


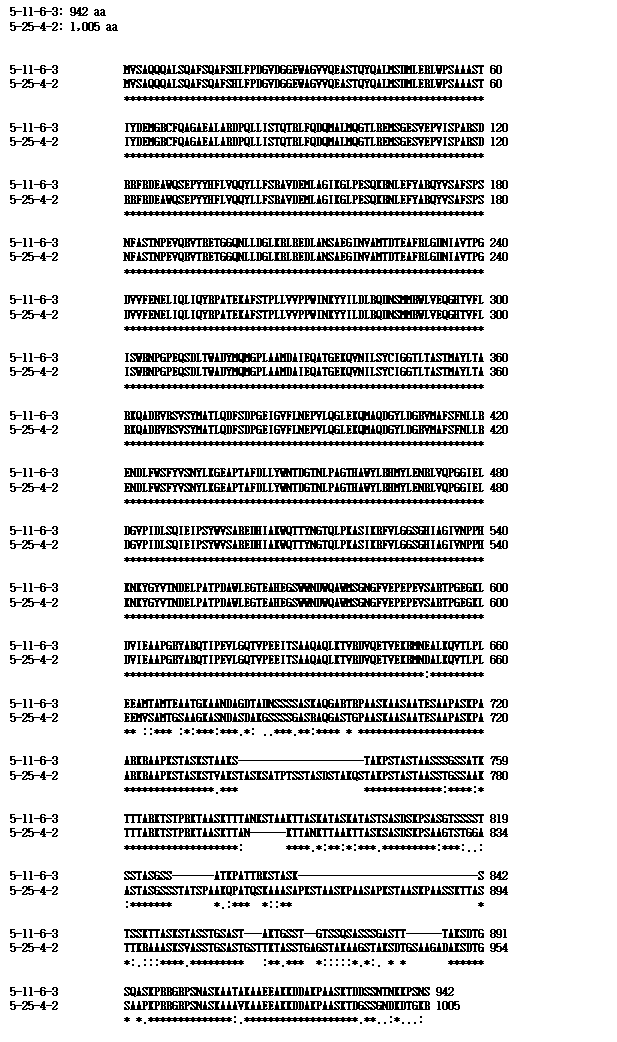
Figure S1 Alignment of the deduced amino acid sequences of Class I PHA synthase (PhaC) from *Cobetia* sp. IU180733JP01 (5-11-6-3) and *Cobetia* sp. IU190790JP01 (5-25-4-2). Sequences were aligned by using clustalW (Thompson et al., 1994).

Reference

Thompson, J.D., Higgins, D.G., Gibson, T.J. (1994). CLUSTAL W: improving the sensitivity of progressive multiple sequence alignment through sequence weighting, position-specific gap penalties and weight matrix choice. *Nucleic Acids Res* 11, 4673-4680.
