# Supplementary material for: Cobetia sp. Bacteria, Which Are Capable of Utilizing Alginate or Waste Laminaria sp. for Poly(3-Hydroxybutyrate) Synthesis, Isolated From a Marine Environment
Source: Front Bioeng Biotechnol. 2020 Aug 25;8:974. doi: 10.3389/fbioe.2020.00974 (PMC7479843; doi:10.3389/fbioe.2020.00974)
Supplement: Supplementary file 2 [file Data_Sheet_2.DOCX]

**Supplementary Material**

Figure S2 Gel permeation chromatography of poly(3-hydroxybutyrate ) produced by *Cobetia* sp. IU180733JP01 (5-11-6-3) (A) and *Cobetia* sp. IU190790JP01 (5-25-4-2) (B).

A


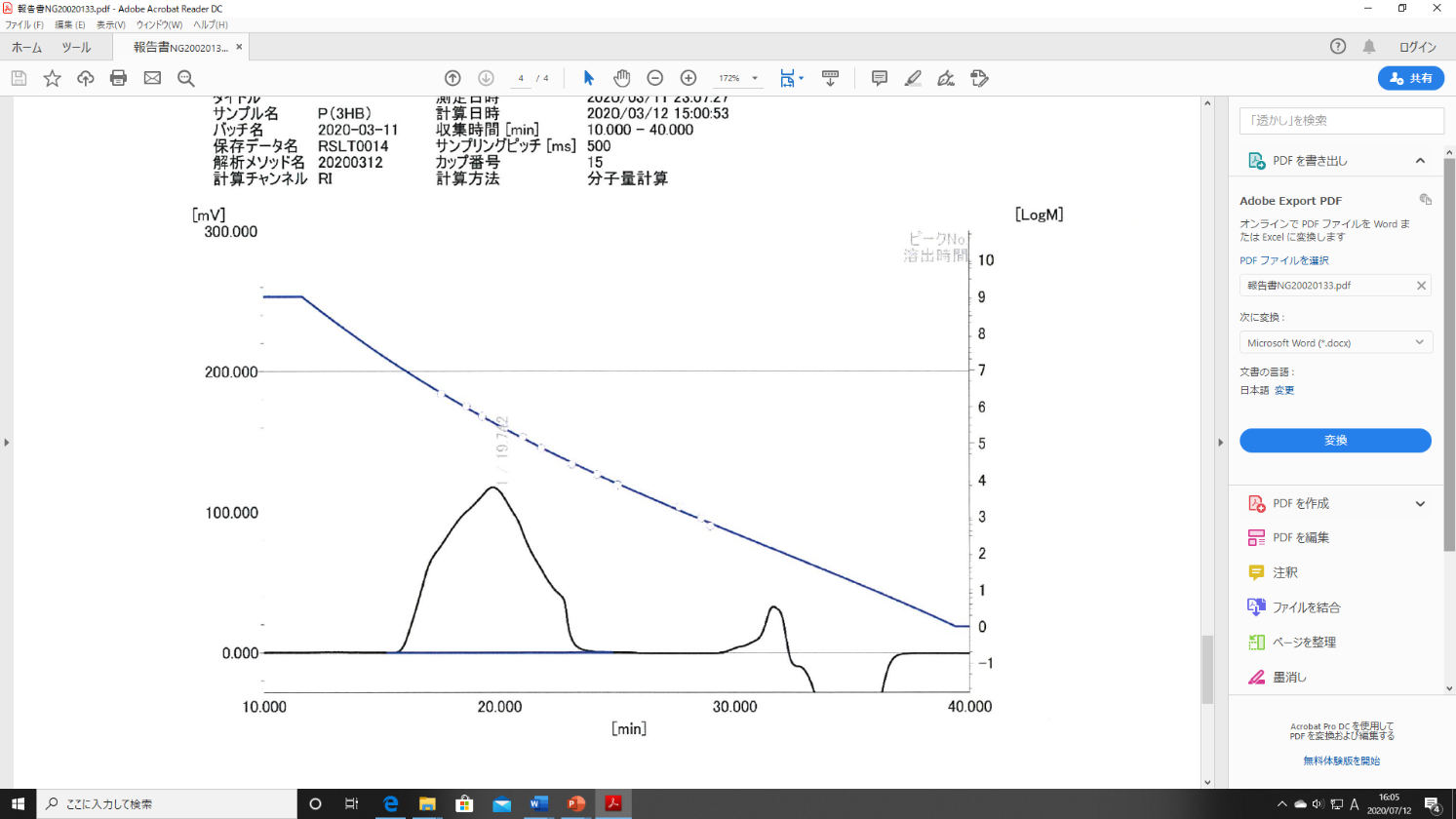


HFIP

B


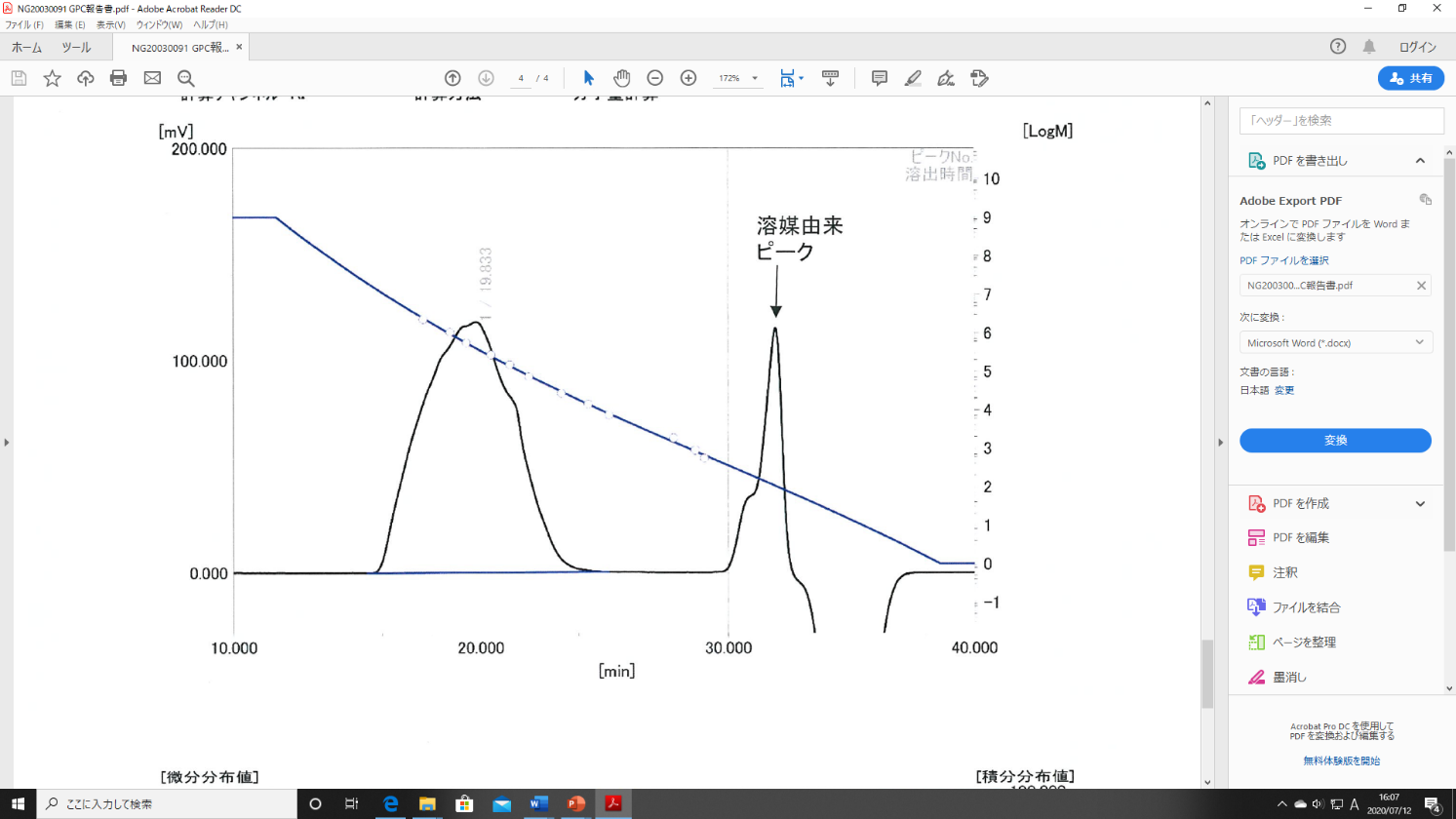


HFIP
